# The Oakville Oil Refinery Closure and Its Influence on Local Hospitalizations: A Natural Experiment on Sulfur Dioxide

**DOI:** 10.3390/ijerph15092029

**Published:** 2018-09-17

**Authors:** Wesley S. Burr, Robert Dales, Ling Liu, Dave Stieb, Marc Smith-Doiron, Branka Jovic, Lisa Marie Kauri, Hwashin Hyun Shin

**Affiliations:** 1 Environmental Health Science and Research Bureau, Health Canada, Ottawa, ON K1A 0K9, Canada; wesleyburr@trentu.ca (W.S.B.); r.dales@canada.ca (R.D.); ling.liu@canada.ca (L.L.); dave.stieb@canada.ca (D.S.); marc.smith-doiron@canada.ca (M.S.-D.); branka.jovic@canada.ca (B.J.); lisamarie.kauri@canada.ca (L.M.K.); 2 Ottawa Hospital Research Institute, University of Ottawa, Ottawa, ON K1H 8L6, Canada; 3 School of Epidemiology and Public Health, University of Ottawa, Ottawa, ON K1G 5Z3, Canada; 4 Department of Mathematics and Statistics, Queen’s University, Kingston, ON K7L 3N6, Canada

**Keywords:** natural experiment, air pollution, sulfur dioxide, respiratory hospitalization, standardized hospitalization ratio

## Abstract

*Background*: An oil refinery in Oakville, Canada, closed over 2004–2005, providing an opportunity for a natural experiment to examine the effects on oil refinery-related air pollution and residents’ health. *Methods*: Environmental and health data were collected for the 16 years around the refinery closure. Toronto (2.5 million persons) and the Greater Toronto Area (GTA, 6.3 million persons) were used as control and reference populations, respectively, for Oakville (160,000 persons). We compared sulfur dioxide and age- and season-standardized hospitalizations, considering potential factors such as changes in demographics, socio-economics, drug prescriptions, and environmental variables. *Results*: The closure of the refinery eliminated 6000 tons/year of SO_2_ emissions, with an observed reduction of 20% in wind direction-adjusted ambient concentrations in Oakville. After accounting for trends, a decrease in cold-season peak-centered respiratory hospitalizations was observed for Oakville (reduction of 2.2 cases/1000 persons per year, p=0.0006) but not in Toronto *(p* = 0.856) and the GTA *(p* = 0.334). The reduction of respiratory hospitalizations in Oakville post closure appeared to have no observed link to known confounders or effect modifiers. *Conclusion*: The refinery closure allowed an assessment of the change in community health. This natural experiment provides evidence that a reduction in emissions was associated with improvements in population health. This study design addresses the impact of a removed source of air pollution.

## 1. Introduction

A very large number of population-based observational studies have reported a positive association between ambient concentrations of air pollution and hospitalizations for respiratory and cardiac disease; however, there exist few studies assessing the health benefit of an intervention which reduces community exposure to air pollution. Improvements in both air quality and health effects have been documented following a number of large-scale changes in local air pollution emissions, including copper smelters in the Southwestern United States [1], bitumous coal burning in Dublin, Ireland [2], lowered sulfur content for fuel oil and gasoline in Hong Kong [3], closure of pollution-emitting industries in East Germany [4], reduction of traffic during the 1996 Summer Olympic Games in Atlanta [5], and reduction of traffic during the Summer Asian Games in Busan, South Korea [6]. Studies have also been done following the closure of the Utah Valley Steel Mill [7,8] and on the impact of the air pollution-lowering steps adopted prior to the 2008 Beijing Olympics [9,10]. In many of these studies the intervention led to significant reductions in negative health effect metrics for the affected populations.

The present study assesses the health impact of the closure of an oil refinery in Oakville, Canada on residents’ hospitalizations. The refinery was designed for production of consumer-grade gasoline and by-products such as asphalt and kerosene. It was located near the shore of Lake Ontario and produced 13,200 cubic meters of gasoline per day (approximately 90,000 barrels) [11]. The refinery was built in 1958 [12] and closed and decommissioned over a six-month period from October 2004 to March 2005. There remains a small-scale oil terminal at the location, but air pollution emissions, in particular SO_2_, have been almost completely eliminated after the refinery closure. Ambient measurements of air pollution for the city were more complete than site-specific emissions data for the study period, as the site-specific monitor was removed before the refinery closure. Considering the relatively small population size of Oakville, we examined respiratory-related morbidity, as there were more hospital admissions than deaths, providing more statistical power. Thus, in this study we focused on the changes in ambient air pollution in relation to those in morbidity records to measure the impact of the refinery closure on public health.

## 2. Materials and Methods

### 2.1. Data

All data referenced in the following section were recorded in, and around, the city of Oakville, Ontario (ON), Canada. Oakville sits on the northwestern shore of Lake Ontario, one of the five great lakes, and experiences a humid continental climate, with cold winters and warm summers. Typical monthly average temperatures range from −5 °C to +26 °C. The city itself is largely suburban, and major pollutant sources nearby include the city of Hamilton, ON (which has a large industrial region) and prevailing winds carrying pollution from the Ohio River valley of the United States up through Southwestern Ontario. No major changes to pollution sources in the local region occurred during the study period aside from the closure of the refinery described in the following, excepting only the national regulatory change (enacted in 2002–2003) which lowered the sulfur content of gasoline: by early 2003 this change was fully in effect. For comparisons with neighbor cities during the study period, City of Toronto (Toronto) was selected as a control city and the Greater Toronto Area (GTA) was used as a population demographic reference to more accurately represent the demographic composition of Oakville.

#### 2.1.1. Emissions Data

To address the emission reduction from closure of the refinery, we obtained site-specific emissions data from the National Pollutant Release Inventory (NPRI) program, run by Environment and Climate Change Canada (ECCC). This program collects information from industrial, commercial, and institutional facilities that meet certain minimum emission requirements [13,14]. Data were obtained for air emissions from the refinery of SO_2_, CO, NO_2_, total PM, PM_10_, and PM_2.5_ for 2002–2005 as well as volatile organic compounds (VOCs) [13] for 2002–2012 from both the refinery and the storage facility that replaced it in 2006.

#### 2.1.2. Ambient Air Pollution Data

**Figure 1:**
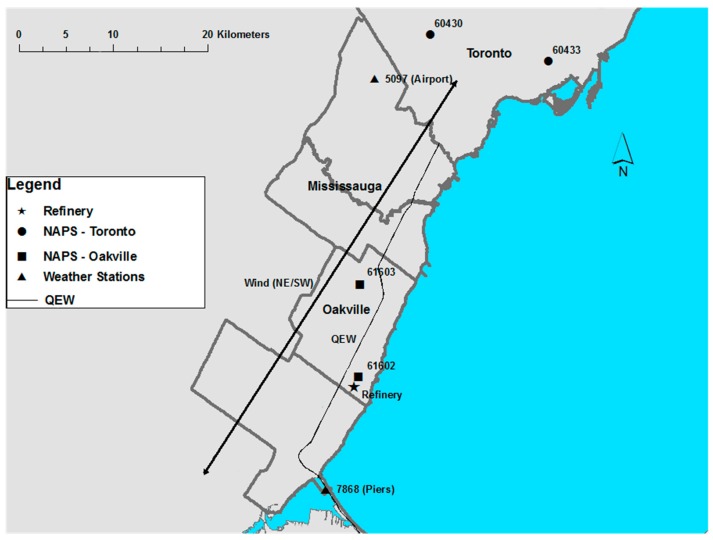
Map of the refinery and surrounding cities with NAPS monitoring stations and weather stations noted. The figure includes a prevailing wind vector, accounting for approximately 40% of all hourly wind directions recorded between the two weather stations, 7868 (Piers) and 5097 (Pearson).

To assess the impact of refinery closure on ambient air pollution, we collected data from two National Air Pollution Surveillance (NAPS) air quality monitoring stations: Station 61602 (approximately 1 km from the refinery site, hereafter called the “Refinery station”) and Station 61603 (approximately 10 km north-east of the refinery, hereafter called the “Oakville station”). Daily values of sulfur dioxide, the indicator pollutant for the oil refinery emissions, were available for 1996–2002 at the Refinery station, and for 2003–2007 at the Oakville station. The Refinery station was closed in 2002 and the Oakville station was opened as a replacement in 2003. Figure 1 shows the two designated sites in the refinery region for the time period of interest, as well as a few stations from surrounding areas, including two NAPS stations (Stations 60430 and 60433) in Toronto and one station (63001) to the southwest in the city of Burlington, ON. The Refinery and Oakville stations in Figure 1 recorded air pollutant monitoring data for some portion of the study period (1996–2012), although no one station has complete data for the entire period. Table 1 gives an overview of the available data from the two designated stations.

#### 2.1.3. Weather Data

Hourly temperature and climate records were obtained from the Climate Data records of ECCC for the study period (1996–2012). These records are almost complete (greater than 99% availability) for two weather stations in the geographic vicinity of the refinery location: one located at Lester B. Pearson International Airport (station 5097 approximately 20 km northeast (NE) of the refinery) and the second located at the Burlington Piers (station 7868, approximately 10 km southwest (SW) of the refinery, as shown in Figure 1). These hourly records allowed us to categorize the prevailing wind patterns through Oakville for the study period. 

#### 2.1.4. Health Data

Daily morbidity (hospitalization) counts for Oakville, Toronto and the GTA were collected for the study period. The International Classification of Diseases 10th Revision (ICD-10) [15] was used to identify circulatory (I00–I99) and respiratory (J00–J99) diagnoses. Ontario hospitals were transitioning between the ICD-9 and ICD-10 classifications over the period 2001–2004, so data recorded under ICD-9 were converted to ICD-10 based on the conversion table provided by the Canadian Institute for Health Information (CIHI).

#### 2.1.5. Additional Data

To investigate potential confounders or effect modifiers related to morbidity we obtained age, sex, marital status, household type, and income from Statistics Canada [16,17] at the census division (CD) level, measured every five years. Standardization was done against the census results of 2006, as a convenient center point of the analysis. Seasonal traffic patterns for the study period on the major highways running through Oakville were also collected to examine their effects on ambient air pollution concentrations and hospitalizations [18].

### 2.2. Methods

#### 2.2.1. Season- and Age-Standardized Hospitalization Rates (SHRs)

In this study, it was necessary to control for seasonal and demographic changes in hospitalization counts when attempting to identify any possible association between reductions in ambient air pollution and hospitalizations for Oakville. For this purpose, a standardized hospitalization rate (SHR) was applied to standardize the number of hospitalizations in Oakville (“observed”) relative to the number of hospitalizations expected for a population with demographic distribution of the GTA in 2006 (“expected”). Seasonal (four 3-month blocks, seasons 1 to 4) aggregate counts were standardized against a distribution of six age categories, as described below, using GTA demographic data for the year 2006 (a census year in Canada). The GTA had a population of 6.3 million people in 2006, and encompasses Oakville, which comprises only a small portion of the overall population (approximately 3%). 

Multiple standardization methods are possible [19,20,21], and direct age-standardization against the reference population of the GTA in 2006, a census year, was selected as the most appropriate method for our available data and demographics. We used six age categories (years): 1–5, 6–20, 21–65, 66–75, 76–85, and 86 or older. We explored variations on this choice of stratification and found that the choice did not affect the analysis; the specific categories chosen here are a convenient choice commonly used at Health Canada for other studies and analyses. 

In the following equation, the SHRs, (*m_t_*), are aggregated by block as follows:

(1) $$m_{t}=\frac{1}{P}\overset{6}{\underset{k=1}{\sum }}\frac{n_{k,t}}{P_{c}}P_{s}$$

where *t* is the time index, *k* the age category (k=1, …, 6), $n_{k,t}$ the city-specific age category health count at given time *t*, $P_{c}$ the city-specific age category population for the given time block, $P_{s}$ the reference population for the same age category for the reference time block, and *P* the total reference population (GTA) for the same. In implementation, the standardization was done by blocks (e.g., 3 months), multiplying the resulting seasonal totals by 4000 to become standardized cases per thousand persons per year. 

#### 2.2.2. Cold-Season Peak-Centered Hospitalization

In addition to the season- and age-standardized SHRs described above, which use fixed seasonal time periods (e.g., January to March), we implemented an alternative, dynamic method, which allows one block per year to float temporally according to annual variations. As the yearly cold and respiratory virus epidemic has variable start and termination dates year-by-year and, additionally, correlates with the maximum respiratory hospitalization rates, it is possible to locate the maximum weekly rate of respiratory hospitalization (between September and July, typically occurring in February or March), and then take a 15-week block centered on that maximum week (i.e., 7 weeks prior to, 7 weeks following, and the maximum week). These 15 weeks of age-standardized rates can then be aggregated and scaled to cases per thousand persons per year. We explore this in the following.

The proposed dynamic approach has value for our analysis over the traditional seasonal approach with a pre-determined fixed time period for two reasons. First, epidemic-related unusual hospitalizations can be accounted for if they exist. Second, off-season epidemic episodes can be accounted for as the dynamic approach puts weight on exact episode occurrence time rather than on calendar time. We use this *cold-season peak-centered* morbidity method to compare Oakville to Toronto. These results, aggregated and simplified to a single count (as a rate per thousand persons per year), were then fit using a simple segmented regression model as follows:

(2) $$R_{t}=\beta_{0}+\beta_{1}t+\beta_{2}I_{t}$$

where $R_{t}$ is the yearly rate, *t* a time variable (year), and $I_{t}$ an indicator variable for the intervention, with the β elements coefficients of the same. This model allows for a long-term trend to be fit to the data while simultaneously modeling any step-function behaviour as delineated by the intervention variable ($I_{t}$*)*. This is a particularly simple implementation of interrupted time-series modeling.

## 3. Results

### 3.1. Changes in Emissions and Overall Ambient Air Pollution 

Table 2 lists the reported total yearly emissions (in metric tons) for the Petro-Canada Refinery (2002–2005) and the Suncor Storage Facility which replaced it (2006–2012). The closure of the refinery in 2004–2005 reduced emissions of SO_2_, CO, NO_2_, PM, and volatile organic compounds (VOCs) by considerable amounts. Annual reported emissions of SO_2_ were reduced from 6000 tons in 2002 to 900 tons (15%) in 2005, corresponding to the three months of operation in calendar year 2005, and then from there to below the reporting threshold. PM emissions dropped from 300–400 tons per year to essentially zero, and VOC emissions were decreased from 600 to 250 tons (42%) immediately after the refinery closure and then further down to approximately 30 tons (5%) in 2012. Blanks in Table 2 indicates emissions below the required reporting thresholds.

In addition to the reported reduction in annual emissions, we observed ambient SO_2_ concentrations as decreasing across the refinery closure period. The SO_2_ fuel regulations initiated in 2003 can be observed in the reduction in annual average hourly ambient SO_2_ for Oakville of 39% (from 4.30 ppb to 2.62 ppb between 2001–2002 and 2003–2004) before the refinery closed. In an attempt at identifying any further reduction that might plausibly be related to the refinery closure in 2004, we compared two time periods, 2003–2004 versus 2005–2007, restricting attention to wind direction from the south-west to allow for winds to carry refinery emissions to station 61603. For this data, the wind direction-restricted average hourly ambient SO_2_ fell further by 20% after the refinery closure (from 4.92 ppb to 3.94 ppb). Against this, the ambient, unrestricted ambient concentration dropped 0.4 ppb, from 2.62 ppb to 2.26 ppb. This larger reduction for the sub-case of wind from the south-west (10% of observed hourly samples) may reflect the impact of the refinery closure. In addition, it appears that the variability of the exposure in both stations decreased, indicating that extreme events were less likely to occur post-closure. Table 3 gives means (µ) and standard deviations (s) for N available hourly observations of both all cases and wind-direction-restricted data (winds originating from the southwest) for the two periods.

### 3.2. Changes in Season- and Age-Standardized Hospitalization Rates (SHRs)

The unstandardized (whether by age or by season) hospital admission data showed little annual daily change in non-accidental all-cause, circulatory and respiratory gross number of hospitalizations over the study period (1996–2012) in Oakville (Table 4). 

**Figure 2:**
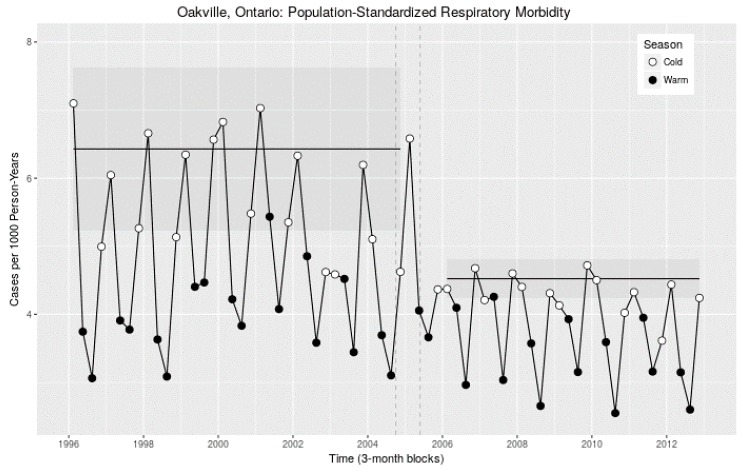
Seasonal (three-month block) population age-standardized total respiratory morbidity (hospitalization) rates (per 1000 persons per year) for Oakville from 1996 through 2012. Cold seasons are displayed with open circles and warm seasons with filled circles. Vertical dashed lines indicate the period during which the refinery was decommissioned, in two stages. The horizontal lines and shaded regions are the cold-season-only means and corresponding 95% confidence intervals for the pre- and post-closure periods.

However, the unstandardized hospital admissions in Table 4 do not reflect demographic (e.g., changes in age distribution and population) or environmental changes (e.g., seasonal variations). Accordingly, we age-standardized the seasonal counts for Oakville against the GTA as a 6.3-million-person demographic reference population, from the national census in 2006 (see Online Supplement, Table S1 for details on the GTA records). As the population of Oakville increased from 132,234 to 190,902 (44%) [22] during the study period, to account for shifting demographics over time we employed age-standardized SHRs to detect any changes in the number of hospitalizations after the refinery closure. In Oakville, respiratory hospitalization rates were up to 50% greater in the cold season (winter, January–March, and fall, October–December, in white dots, Figure 2) than in the warm season (spring, April–June, and summer, July-September, in dark dots, Figure 2), and thus we examined seasonal-level age-standardized SHRs. 

There was a sharp decline in Oakville seasonal age-standardized SHRs of respiratory hospitalizations after the closure of the refinery (Figure 2, to the right of two vertical dashed lines). The 2003 rates appeared to be outliers, which could be explained by the severe acute respiratory syndrome (SARS) epidemic [23] in Ontario when patients were advised to stay away from hospitals. Apart from this specific cold season in 2003, there were approximately 6.5 fall/winter hospitalizations per thousand persons per year before the refinery closure (with the pattern going back as far as 1996, the first year of available data), dropping to a rate of 4.5 immediately following the closure, a 31% decline in respiratory SHRs (mean before to mean after).

To examine the standardization methodology, we explored a number of standardization approaches, including population standardization (year-by-year evolving reference) and static reference population (presented in this paper, e.g., Figure 2). In all cases, the decrease in Oakville stood out as being unusual among nearby cities, and against the demographic reference population of the GTA in 2006. Selected figures from this exploration for Oakville, the City of Toronto, and for the entire GTA are available in the Online Supplement for this paper as Figures S1 through S9.

### 3.3. Changes in Cold-Season Peak-Centered SHRs

**Figure 3:**
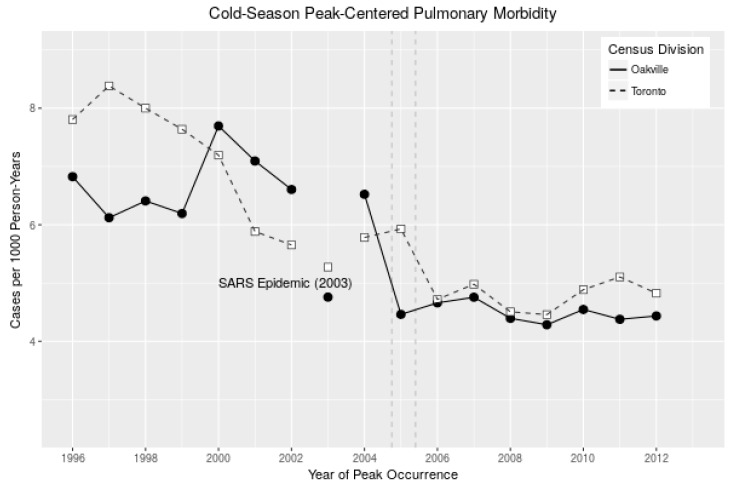
Cold-season peak-centered respiratory hospitalization rates (in cases per 1000 persons per year) for Toronto (dashed, *not* the Greater Toronto Area (GTA)) and Oakville (solid) from 1996 to 2012. Note: 2003 as an outlier due to SARS, and the downward trend of Toronto from 1999 to 2004.

The peak of the respiratory-related hospitalization rates typically occurs during the yearly epidemic of cold and respiratory virus infections, which has variable timing and therefore is inconsistent from city to city across different hospitalization-cause categories. We thus considered a dynamic approach of taking 15-week blocks centered on the peak week of respiratory hospitalizations for a given cold season (October–March) and city. Eliminating 2003 as a clear outlier (attributed to the SARS epidemic) and excluding 2005 as the pivot year for refinery closure, we found a significant (p=0.0006) mean difference between the period before the closure (1996–2004) and afterwards (2006–2012) using a simple segmented linear regression model, as detailed in Equation (2) (details in Table 5 and Figure 3). We found no significant differences in all-cause (non-accidental), circulatory and non-cardiorespiratory related hospitalizations before and after the refinery closure (not shown here). Note that when data from 2003 and 2005 were included in the analysis, although the exact values changed, the conclusions did not; similarly, allowing 2005 to be considered as “after” closure did not change the conclusions. This result indicates that our finding on the Oakville respiratory hospitalizations is insensitive to the inclusion of the two specific years in question. In summary, cold-season peak-centered respiratory hospitalizations in Oakville fell by 2.2 cases per thousand persons per year (approximately 180 total hospitalizations for the year) from before the refinery closure to after, excluding the two outlier years. Across the same period, no immediate decrease (after accounting for trend) was found in Toronto. 

To test the sensitivity of the results to the time of analysis, we repeated the above analysis by iterating through break-point years of 2001 through 2007, again excluding 2003 and 2005 from the analysis. The most significant break-point, as determined by the *p*-value of the segmented regression, occurred in 2004–2005 (Oakville) and 2001 (Toronto). 

### 3.4. Other Possible Confounders

Other potentially confounding factors include changes in socioeconomic variables, temperature and traffic density occurring in the Oakville area over the same time as the refinery closure. However, using census data from 2001, 2006 and 2011 [24], we found no corresponding change in median income, sex, education status, labor force status, or industry of main employment, nor was any such change reported by community organizations [22,25]. For vehicular traffic, the time period of interest shows a steady increase in traffic volume and density, which we cannot plausibly link to a sudden decrease in respiratory-related hospitalizations.

## 4. Discussion

In this study, we found that the closure of the oil refinery located in the city of Oakville, Ontario was associated with measurable reductions in wind-direction-adjusted ambient SO_2_ in Oakville. We additionally found that there was a significant reduction in respiratory-related hospitalizations in Oakville, with the reduction occurring sharply after the refinery closure and persisting. Moreover, we found no other explanatory or confounding factors which influenced the reduction in hospitalizations for Oakville, such as changes in socio-economic status, traffic counts, or traffic-related air pollutants such as NO_2_. Taking these findings together, the reduction in SO_2_ emissions from the Oakville refinery closure and the subsequent decrease in SO_2_ concentrations in ambient air concentrations appear to have occurred simultaneously with a significant reduction in cold-season respiratory hospitalizations in Oakville. 

Natural experiments represent a powerful tool in establishing a causal link between exposure and response. In many cases, the experiment acts as an intervention, with correspondingly large changes: the Utah Valley steel mill closures [7] being one of the most clear and famous examples. In our study the evidence suggests that even closures of smaller-scale industrial sites like the Oakville refinery, if the sites are emitters of pollutants associated with health effects, can have immediate and measurable effects on the health of the surrounding community. 

There were several limitations of our study. Data were lacking from the Refinery Station (61602), which was closed at the end of 2002, and the replacement Oakville station (61603) did not begin operation until April 2003, although data from the NPRI indicated that there was little change in the emissions from the refinery up to 2003, so the 1996–2002 span of available data from 61602 should give reasonably accurate estimates of the ambient air quality before closure. Data from Oakville station provided us a view into ambient air quality after closure. However, no SO_2_ data were available after 2007 for the Oakville Station. In addition, VOC data (which would normally be of great interest when examining the emissions from a refinery) were sparse or unavailable for the period of the study.

The NPRI data used as a gauge of pollution emissions from the refinery are not entirely reliable, as the data are self-reported by industry, rather than observed by a third party. In addition, regulations only require coarse aggregate measures. Self-reported data on air pollution emissions are available from 2002–2014, and emissions of specific chemicals (e.g., toluene) are available back to 1994. As well, yearly NPRI reporting requirements, including addition/removal of substances, reporting thresholds and reporting exemptions for specific industrial sectors have changed over time [14,26]. In 2001, new release groupings were created, including on-site pollutant release to air, and 7 criteria air contaminants were added in 2002, including SO_2_, NOx, CO, and VOCs. 

There are no limitations to the health-related data, as they are complete due to mandatory reporting regulations, with complete morbidity data available back to 1996. The seasonal age-standardized SHRs based on the GTA should account for seasonal, demographic and population changes through the study period, and as demonstrated in the Online Supplement, are resistant to variation in parameter choices for the SHR.

By contrast, our study has several strengths. Use of SO_2_ as a proxy for overall refinery emissions is a sensible choice, as SO_2_ is known to be related to morbidity (hospitalizations), both in general [27,28,29,30,31,32] and specifically for the Canadian population [33,34]. While associations between SO_2_ and morbidity are positive and significant, they are small in magnitude, so any association between refinery closure and health outcomes cannot be necessarily directly attributed to this pollutant, however use of SO_2_ as a representative proxy of the whole seems reasonable. At the same time, no other ambient air pollutants with available data showed statistically significant reductions of step-function nature after the refinery closure, despite the clear reduction (6000 tons per year for SO_2_, Table 2) in emissions, so again, the use of SO_2_ is justified.

The closure of the refinery coincided with a measurable reduction in hourly ambient SO_2_ levels in the city of Oakville—a reduction that was greater than what would be expected from the refinery closure, which we estimated through evaluation of similar monitoring stations in the neighboring city of Toronto using wind-direction-restricted analyses. Comparing the before- and after-closure periods, we found a statistically significant (step-function) reduction of ambient SO_2_ concentration levels in Oakville only, not in Toronto. This is another strength of the study, as with the local nature of SO_2_ ambient concentration we would not expect to see step-function reductions of SO_2_ concentration outside of the immediate region surrounding the refinery. 

The closure of the refinery came soon after the introduction of a stringent sulfur content regulation for gasoline in Ontario: as of 1 January 2005, all gasoline sold in Ontario was limited to an annual average sulfur level of 30 ppm (30,000 ppb) or 30 milligrams per-kilogram (mg/kg), with a never-to-be-exceeded limit of 80 ppm (Government of Canada, 1999). From July 2002 to 2005, there was an interim annual average sulfur content of 150 ppm. The ambient concentration reduction happened quite sharply in 2002–2003, despite the regulation rolling out in phases, as most fuel manufacturers moved their facilities to the new standard in time for the initiation of the regulation. Similar regulatory changes were implemented in June 2006 for diesel fuel, with a reduction from 500 to 15 ppm [35]. These changes, together with more restrictions on power plants and industry, led to a decrease in the Ontario average annual ambient concentration of sulfur dioxide (SO_2_) from approximately 6 ppb to 2.2 ppb across the 2000–2013 period [36,37]. The step-wise decrease in ambient SO_2_ levels observed in Oakville appears to be in addition to the background decrease in SO_2_ levels associated with the reduction in allowable sulfur content in gasoline, as seen in ambient concentrations of nearby municipalities.

Regarding other commonly available air pollutant measures, NO_2_ concentrations have also been previously linked to morbidity [33,34]; however, examination of the present data revealed neither a significant decrease in 2001–2003 such as was observed in SO_2_ with the roll-out of the Canadian Environmental Protection Act (1999) [35] regulations, nor any sudden decrease in Oakville levels at or around the closure of the refinery. As NO_2_ is most commonly linked to automobile traffic, this fits available evidence, supporting the NPRI records which indicate that NO_2_ was not a significant emission from the refinery.

While the Census Division of Halton (which contains CSD Oakville) experienced rapid population growth through the 2001–2011-time period, much of the growth occurred in newly-built subdivisions well north of Lake Ontario, away from the refinery and north of the highway system [22,25]. The area directly co-located with the refinery saw very little population growth (0–3%) during this time. In addition, the age distribution skewed toward retirees through this period, with the demographic profile aging over the decade; much of this aging should have been compensated for by the standardization of hospitalization records. Any residual effect not compensated for by this modeling (due to population demographics being temporally coarse owing to census patterns) should have been in the direction of an aging population, which should result in increased levels of hospitalizations rather than the opposite.

In conclusion, despite limitations in data availability for the region and the study, we have demonstrated measurable reductions in ambient SO_2_ levels as would be experienced by the residents of Oakville. We similarly demonstrated a sharp decrease in cold-season age-standardized hospitalization rates, which occurred immediately following the refinery closure. No other contributory factor was found which could explain this decrease. Thus, we present in this study another piece of evidence in the field, showing concurrent measurable reductions in both SO_2_ concentrations and respiratory-related hospitalizations when a local emission source was removed.

## 5. Conclusions

This study demonstrated improved air quality and population health ameliorated in relation with the closure of an oil refinery. As a natural experiment, it provides evidence supporting a link between refinery emissions and adverse health effects.

## Figures and Tables

**Table 1:** National Air Pollution Survey (NAPS) data availability from monitoring stations near the refinery (61602 and 61603) in Figure 1.

NAPS ID	Air Pollutant	Start Date	End Date	Availability
Refinery station 61602	SO_2_	01 January 1996	31 December 2002	93.6%
	CO	01 January 1996	31 December 2002	96.6%
	NO_2_	01 January 1996	31 December 2002	92.1%
	PM_2.5_	NA		
	O_3_	01 January 1996	31 December 2002	94.8%
Oakville station 61603	SO_2_	28 April 2003	31 December 2007	75.9%
	CO	29 April 2003	31 December 2004	94.3%
	NO_2_	29 April 2003	31 December 2011	97.5%
	PM_2.5_	29 May 2003	31 December 2011	98.6%
	O_3_	29 April 2003	31 December 2011	98.4%

**Table 2:** Annual changes in emissions from the refinery: 2002–2012 (in tons). VOCs: volatile organic compounds.

Facility	Year	SO_2_	CO	NO_2_	PM	PM_10_	PM_2.5_	VOCs
Petro-Canada Refinery	2002	5984	277	626	318	224	139	558
	2003	5581	239	606	434	315	189	568
	2004	4615	277	700	436	303	176	605
	2005	933	61	162	82	58	34	252
Suncor Storage Facility	2006							124
	2007							149
	2008							116
	2009							116
	2010							29
	2011							29
	2012							33

**Table 3:** Wind-adjusted ambient SO_2_ concentration data in ppb for the Oakville, 61603. The wind-direction restriction used a 90-degree arc (wind from the southwest (SW), 180–270°, directed toward the northeast, 0–90°), coarsely aligned with the N$14^{{}^{\circ}}$E path joining the two weather stations, and including the path of wind from the refinery to station 61603 in Northeast Oakville.

Year	NAPS ID	All Data			Restricted—From SW		
		μ	*s*	*N*	μ	*s*	*N*
2003–2004	61603	2.62	3.93	13821	4.92	5.03	1402
2005–2007		2.26	3.03	17254	3.94	4.58	1689

**Table 4:** Summary statistics for daily counts of hospitalizations by cause in Oakville for 1996–2012.

Year	Daily All-Cause ^a^			Daily Circulatory ^b^			Daily Respiratory ^c^		
	Mean	SD ^d^	Max ^e^	Mean	SD	Max	Mean	SD	Max
1996	19.2	6.2	36	3.4	2.0	10	1.6	1.4	7
1997	19.1	5.9	37	3.3	1.9	10	1.6	1.4	9
1998	19.5	6.5	41	3.3	1.8	10	1.6	1.4	6
1999	20.5	6.1	36	3.3	1.9	9	2.0	1.6	9
2000	20.2	6.1	40	3.1	2.0	13	1.9	1.5	9
2001	21.7	6.7	38	3.2	1.9	11	2.1	1.5	9
2002	20.8	6.3	40	3.1	1.9	9	1.9	1.5	8
2003	21.1	6.2	38	3.0	1.8	10	1.9	1.6	10
2004	20.8	6.1	39	3.0	1.9	10	1.8	1.4	9
2005	22.0	6.5	43	3.1	1.8	8	2.1	1.6	8
2006	21.6	6.4	38	3.0	1.9	9	1.9	1.4	7
2007	20.4	6.0	41	2.6	1.7	10	2.0	1.4	7
2008	19.6	6.1	47	2.3	1.5	7	1.9	1.4	7
2009	21.1	6.0	38	2.6	1.6	8	2.1	1.5	8
2010	20.7	6.1	37	2.7	1.8	8	2.0	1.5	8
2011	21.4	6.1	38	2.6	1.6	8	2.1	1.6	8
2012	20.7	6.1	38	2.7	1.6	8	2.1	1.5	6

^a^ non-accidental (ICD-10, A00-R99); ^b^ ICD-10, I00-I99; ^c^ ICD-10, J00-J99. ^d^ standard deviation; ^e^ annual maximum of daily maximum counts.

**Table 5:** Difference in means (in ppb) before and after 2005, for cold- and virus-peak-centered hospitalizations, using segmented regression via Equation (2).

ICD Cause	Oakville *		Toronto *	
	$\;\beta_{2}$	*p*-Value	$\;\beta_{2}$	*p*-Value
All-cause (1)	−5.1	0.0816	2.3	0.5291
Circulatory (2)	−0.8	0.3071	0.3	0.6386
Respiratory (3)	−2.2	0.0006	−0.1	0.8564
Non-cardiorespiratory (1 − (2 + 3))	−3.9	0.0911	2.2	0.4257

* Years 2003 and 2005 are excluded from the model as outliers; inclusion does not change the conclusion.

## Supplementary Materials

The following are available online at http://www.mdpi.com/1660-4601/15/9/2029/s1, Figure S1: Oakville seasonal (3-month blocks) non-age-standardized total respiratory hospitalizations from 1996 through 2010; Figure S2: Oakville seasonal (3-month blocks) year-by-year age-standardized total respiratory hospitalization rate from 1996 through 2010, in 1000-persons per year. Standardization done against Oakville population for given year (evolving over time); Figure S3: Oakville seasonal (3-month blocks) population age-standardized total respiratory hospitalization rate from 1996 through 2010, in 1000-persons per year. Standardization done against a fixed population distribution for the GTA in 2006, a census year; Figure S4: Toronto seasonal (3-month blocks) total non-age-standardized total respiratory hospitalizations from 1996 through 2010; Figure S5: Toronto seasonal (3-month blocks) year-by-year age-standardized total respiratory hospitalization rate from 1996 through 2010, in 1000-persons per year. Normalization done against Toronto population for *given* year (evolving over time); Figure S6: Toronto seasonal (3-month blocks) population age-standardized total respiratory hospitalization rate from 1996 through 2010, in 1000-persons per year. Standardization done against a fixed population distribution for the GTA in 2006, a census year; Figure S7: GTA seasonal (3-month blocks) non-age-standardized total respiratory hospitalizations from 1996 through 2010; Figure S8: GTA seasonal (3-month blocks) year-by-year age-standardized total respiratory hospitalization rate from 1996 through 2010, in 1000-persons per year. Standardization done against the current GTA population for given year (evolving over time); Figure S9: GTA seasonal (3-month blocks) age-standardized total respiratory hospitalization rate from 1996 through 2010, in 1000-persons per year. Standardization done against a fixed population distribution for the GTA in 2006, a census year; Table S1: yearly mean, standard deviation and maximum counts (with daily resolution) for all-cause, circulatory and respiratory hospitalizations for the GTA, a region encompassing Toronto, Ontario as well as a number of other co-located cities.
