# Supplementary material for: The Oakville Oil Refinery Closure and Its Influence on Local Hospitalizations: A Natural Experiment on Sulfur Dioxide
Source: Int J Environ Res Public Health. 2018 Sep 17;15(9):2029. doi: 10.3390/ijerph15092029 (PMC6163796; doi:10.3390/ijerph15092029)
Supplement: Supplementary file 1 [file ijerph-15-02029-s001.zip › ijerph-344392-supplementary.docx]

Online Supplement for:

The Oakville Oil Refinery Closure and Its Influence on Local Hospitalizations: A Natural Experiment on Sulfur Dioxide

Wesley S. Burr^1^, Robert Dales^1,2^, Ling Liu^1^, Dave Stieb^1,3^, Marc Smith-Doiron^1^, Branka Jovic^1^, Lisa Marie Kauri^1^, and Hwashin Hyun Shin^1,4 *^

1. Environmental Health Science and Research Bureau, Health Canada, Ottawa, ON, K1A 0K9, Canada
2. Ottawa Hospital Research Institute, and University of Ottawa, Ottawa, ON K1H 8L6, Canada

^3^ School of Epidemiology and Public Health, University of Ottawa, Ottawa, ON, K1G 5Z3, Canada

^4^ Dept. of Mathematics and Statistics, Queen's University, Kingston, ON, K7L 3N6, Canada

***** Correspondence: hwashin.shin@canada.ca; Tel.: +1-613-851-3658

In this Online Supplement we present a number of representative figures supporting our explorations into methods of standardizing rates of respiratory hospitalizations. The figures show three selected results for each of Oakville, City of Toronto (Toronto), and the Greater Toronto Area (GTA), all geographically co-located in the “Golden Horseshoe” north/north-west of Lake Ontario in Ontario, Canada. All figures should be compared to Figure 2 of the associated paper for context. The first figure for each geographic area is non-standardized against any demographic distribution; the second is standardized (or normalized may be the more appropriate word) against the population of that area for the given year; and the third is truly standardized, against a standard of the population demographic distribution for the GTA for 2006, a census year. We conclude the supplement with a supplemental table showing summary statistics for the GTA, for contrast with Table S1.


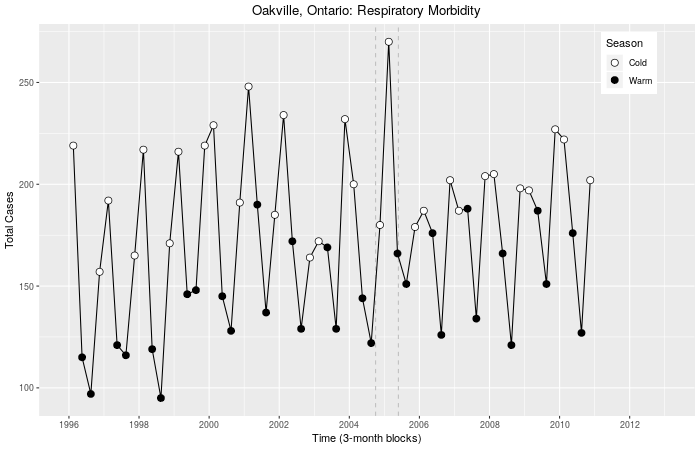


Figure S1: Oakville seasonal (3-month blocks) non-age-standardized total respiratory hospitalizations from 1996 through 2010: (1) open circles for cold seasons; (2) filled circles for warm seasons; and (3) vertical dashed lines for the period of the refinery closure.


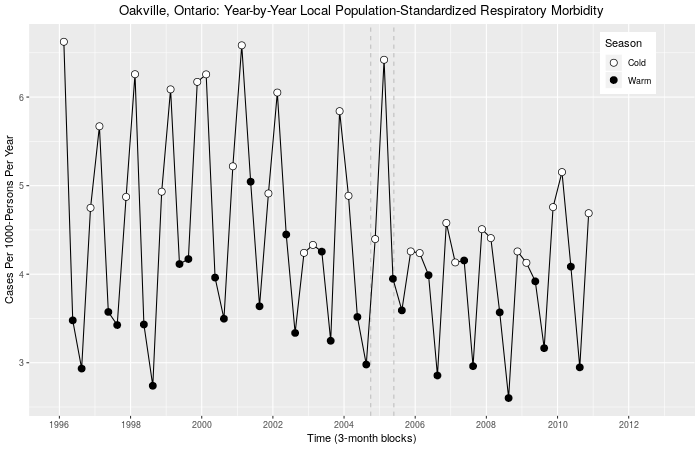


Figure S2: Oakville seasonal (3-month blocks) year-by-year age-standardized total respiratory hospitalization rate from 1996 through 2010, in 1000-persons per year. Standardization done against Oakville population for *given* year (evolving over time): (1) open circles for cold seasons; (2) filled circles for warm seasons; and (3) vertical dashed lines for the period of the refinery closure.


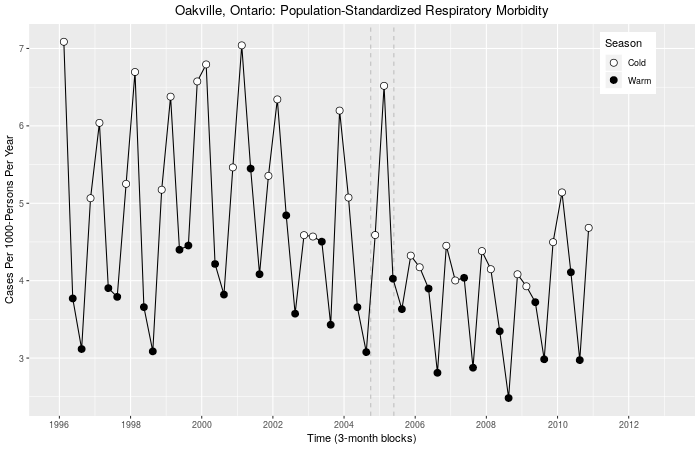


Figure S3: Oakville seasonal (3-month blocks) population age-standardized total respiratory hospitalization rate from 1996 through 2010, in 1000-persons per year. Standardization done against a fixed population distribution for the GTA in 2006, a census year: (1) open circles for cold seasons; (2) filled circles for warm seasons; and (3) vertical dashed lines for the period of the refinery closure. Note: this figure is the same figure, without the explanatory means, as Figure 2 of the main paper, included here for flow and comparison purposes.


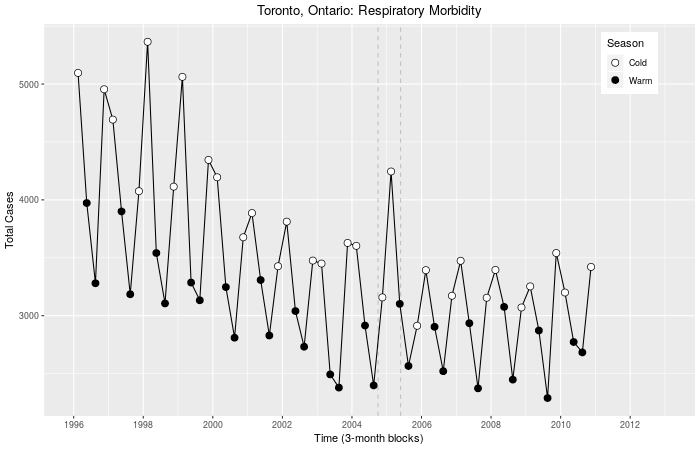


Figure S4: Toronto seasonal (3-month blocks) total non-age-standardized total respiratory hospitalizations from 1996 through 2010: (1) open circles for cold seasons; (2) filled circles for warm seasons; and (3) vertical dashed lines for the period of the refinery closure.


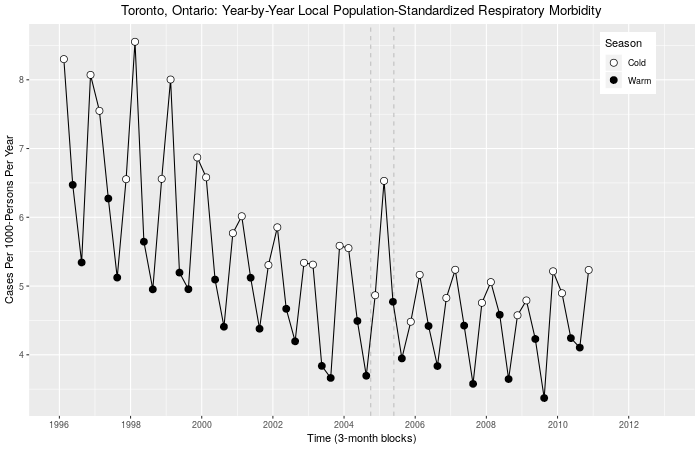


Figure S5: Toronto seasonal (3-month blocks) year-by-year age-standardized total respiratory hospitalization rate from 1996 through 2010, in 1000-persons per year. Normalization done against Toronto population for *given* year (evolving over time) : (1) open circles for cold seasons; (2) filled circles for warm seasons; and (3) vertical dashed lines for the period of the refinery closure.


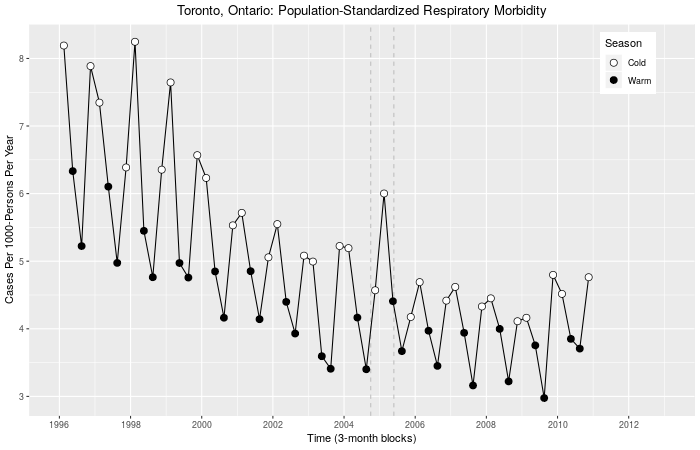


Figure S6: Toronto seasonal (3-month blocks) population age-standardized total respiratory hospitalization rate from 1996 through 2010, in 1000-persons per year. Standardization done against a fixed population distribution for the GTA in 2006, a census year: (1) open circles for cold seasons; (2) filled circles for warm seasons; and (3) vertical dashed lines for the period of the refinery closure.


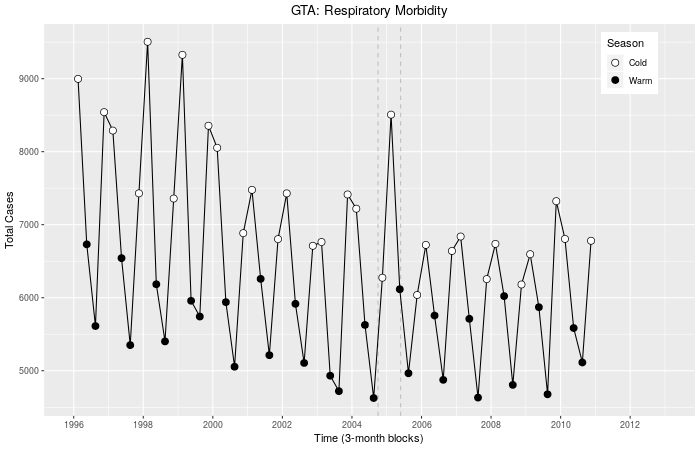


Figure S7: GTA seasonal (3-month blocks) non-age-standardized total respiratory hospitalizations from 1996 through 2010: (1) open circles for cold seasons; (2) filled circles for warm seasons; and (3) vertical dashed lines for the period of the refinery closure.


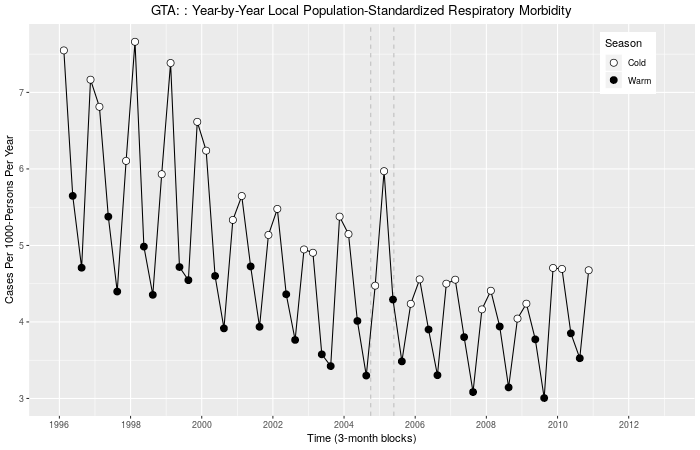


Figure S8: GTA seasonal (3-month blocks) year-by-year age-standardized total respiratory hospitalization rate from 1996 through 2010, in 1000-persons per year. Standardization done against the current GTA population for *given* year (evolving over time): (1) open circles for cold seasons; (2) filled circles for warm seasons; and (3) vertical dashed lines for the period of the refinery closure.


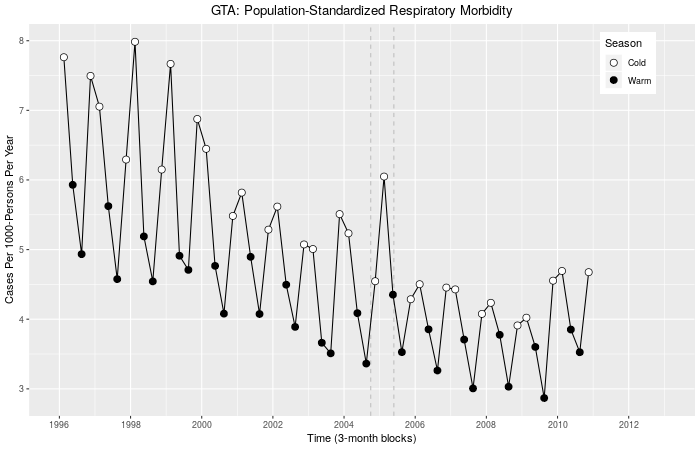


Figure S9: GTA seasonal (3-month blocks) age-standardized total respiratory hospitalization rate from 1996 through 2010, in 1000-persons per year. Standardization done against a fixed population distribution for the GTA in 2006, a census year: (1) open circles for cold seasons; (2) filled circles for warm seasons; and (3) vertical dashed lines for the period of the refinery closure.

Table 4 of the paper presents the yearly statistics for hospitalizations for Oakville, Ontario. The following table, Table S1, presents the complementary statistics for the GTA, for comparison purposes.

**Table S1**: yearly mean, standard deviation and maximum counts (with daily resolution) for all-cause, circulatory and respiratory hospitalizations for the GTA, a region encompassing Toronto, Ontario as well as a number of other co-located cities.

|  | **Daily All-Cause^a^** | | | **Daily Circulatory^b^** | | | **Daily Respiratory^c^** | | |
| --- | --- | --- | --- | --- | --- | --- | --- | --- | --- |
| Year | **Mean** | **SD^d^** | **Max^e^** | **Mean** | **SD** | **Max** | **Mean** | **SD** | **Max** |
| 1996 | 888.9 | 212.8 | 1257.0 | 135.9 | 28.1 | 199.0 | 83.2 | 27.6 | 172.0 |
| 1997 | 856.9 | 199.1 | 1187.0 | 135.5 | 26.9 | 199.0 | 77.3 | 22.2 | 172.0 |
| 1998 | 850.3 | 193.2 | 1174.0 | 132.4 | 25.9 | 185.0 | 79.6 | 25.3 | 167.0 |
| 1999 | 843.4 | 182.3 | 1107.0 | 127.6 | 23.5 | 184.0 | 82.5 | 26.9 | 182.0 |
| 2000 | 820.7 | 174.1 | 1101.0 | 126.3 | 24.7 | 190.0 | 72.7 | 22.0 | 196.0 |
| 2001 | 842.2 | 174.7 | 1100.0 | 127.3 | 26.1 | 188.0 | 72.6 | 16.7 | 128.0 |
| 2002 | 826.1 | 168.5 | 1043.0 | 122.5 | 23.7 | 171.0 | 70.8 | 16.5 | 123.0 |
| 2003 | 780.2 | 169.5 | 1045.0 | 114.4 | 24.7 | 166.0 | 67.2 | 20.4 | 155.0 |
| 2004 | 802.8 | 165.2 | 1039.0 | 117.2 | 24.7 | 176.0 | 66.6 | 16.2 | 123.0 |
| 2005 | 803.1 | 167.3 | 1063.0 | 114.5 | 22.8 | 169.0 | 72.3 | 20.9 | 141.0 |
| 2006 | 776.1 | 156.1 | 1040.0 | 112.8 | 23.3 | 167.0 | 67.6 | 14.9 | 110.0 |
| 2007 | 767.4 | 148.8 | 971.0 | 109.5 | 21.3 | 164.0 | 66.2 | 16.2 | 141.0 |
| 2008 | 765.9 | 147.9 | 986.0 | 110.3 | 21.2 | 162.0 | 66.8 | 15.1 | 121.0 |
| 2009 | 778.4 | 147.4 | 1022.0 | 110.4 | 21.4 | 155.0 | 69.1 | 16.6 | 124.0 |
| 2010 | 789.8 | 148.0 | 1026.0 | 113.2 | 21.2 | 166.0 | 68.5 | 17.0 | 142.0 |

^a^non-accidental (ICD-10, A00-R99); ^b^ICD-10, I00-I99; ^c^ICD-10, J00-J99. ^d^standard deviation; ^e^annual maximum of daily maximum counts.

© 2018 by the authors. Submitted for possible open access publication under the
terms and conditions of the Creative Commons Attribution (CC BY) license (http://creativecommons.org/licenses/by/4.0/).
